# Altered development of dopaminergic neurons differentiated from stem cells from human exfoliated deciduous teeth of a patient with Down syndrome

**DOI:** 10.1186/s12883-018-1140-2

**Published:** 2018-08-31

**Authors:** Thanh Thi Mai Pham, Hiroki Kato, Haruyoshi Yamaza, Keiji Masuda, Yuta Hirofuji, Hiroshi Sato, Huong Thi Nguyen Nguyen, Xu Han, Yu Zhang, Tomoaki Taguchi, Kazuaki Nonaka

**Affiliations:** 1 Section of Oral Medicine for Child, Division of Oral Health, Growth & Development, Faculty of Dental Science, Kyushu University, Maidashi 3-1-1, Higashi-Ku, Fukuoka, 812-8582 Japan; 2 Department of Pediatric Surgery, Reproductive and Developmental Medicine, Graduate School of Medical Sciences, Kyushu University, Maidashi 3-1-1, Higashi-Ku, Fukuoka, 812-8582 Japan

**Keywords:** Down syndrome, Dopamine, Human exfoliated deciduous teeth, SHED, Stem cells, Dopaminergic neurons, Differentiation, Dopamine secretion

## Abstract

**Background:**

Down syndrome (DS) is a common developmental disorder resulting from the presence of an additional copy of chromosome 21. Abnormalities in dopamine signaling are suggested to be involved in cognitive dysfunction, one of the symptoms of DS, but the pathophysiological mechanism has not been fully elucidated at the cellular level. Stem cells from human exfoliated deciduous teeth (SHED) can be prepared from the dental pulp of primary teeth. Importantly, SHED can be collected noninvasively, have multipotency, and differentiate into dopaminergic neurons (DN). Therefore, we examined dopamine signaling in DS at the cellular level by isolating SHED from a patient with DS, differentiating the cells into DN, and examining development and function of DN.

**Methods:**

Here, SHED were prepared from a normal participant (Ctrl-SHED) and a patient with DS (DS-SHED). Initial experiments were performed to confirm the morphological, chromosomal, and stem cell characteristics of both SHED populations. Next, Ctrl-SHED and DS-SHED were differentiated into DN and morphological analysis of DN was examined by immunostaining. Functional analysis of DN was performed by measuring extracellular dopamine levels under basal and glutamate-stimulated conditions. In addition, expression of molecules involved in dopamine homeostasis was examined by quantitative real-time polymerase chain reaction and immunostaining. Statistical analysis was performed using two-tailed Student’s *t*-tests.

**Results:**

Compared with Ctrl-SHED, DS-SHED showed decreased expression of nestin, a neural stem-cell marker. Further, DS-SHED differentiated into DN (DS-DN) exhibiting decreased neurite outgrowth and branching compared with Ctrl-DN. In addition, DS-DN dopamine secretion was lower than Ctrl-DN dopamine secretion. Moreover, aberrant expression of molecules involved in dopaminergic homeostasis was observed in DS-DN.

**Conclusions:**

Our results suggest that there was developmental abnormality and DN malfunction in the DS-SHED donor in this study. In the future, to clarify the detailed mechanism of dopamine-signal abnormality due to DN developmental and functional abnormalities in DS, it is necessary to increase the number of patients for analysis. Non-invasively harvested SHED may be very useful in the analysis of DS pathology.

**Electronic supplementary material:**

The online version of this article (10.1186/s12883-018-1140-2) contains supplementary material, which is available to authorized users.

## Background

Down syndrome (DS) is caused by an extra copy of chromosome 21 and is one of the most common developmental disorders. Reported symptoms of DS include impairment of cognitive functions, such as learning, memory, language, and executive function [1–4]. Dopamine (DA) is an important neurotransmitter in the regulation of cognitive function. It has been suggested that disturbance of the DA signaling system causes the cognitive impairments observed in DS [5–7].

The amount of DA in the brain and cerebrospinal fluid (CSF) of patients with DS has been reported to be both higher and lower than that in healthy people [8, 9], suggesting that a disturbance in DA homeostasis is implicated in DS. It has also been reported that there is reduced expression of DA receptors D1R and D2R in the brains of patients with DS [10]. As observed in patients with DS, varied amounts of DA have also been reported in mouse models of DS [6, 7, 11, 12], suggesting that variable DA levels are associated with abnormal brain development. However, the role of the DA signaling system in DS pathology has yet to be analyzed at a cellular level.

Stem cells from human exfoliated deciduous teeth (SHED) can be acquired noninvasively and used for research [13–15]. Thus, using SHED, consent to participate in research may be obtained more readily from the parents of young patients. SHED can be differentiated into dopaminergic neurons (DN) and used for the treatment of a parkinsonian rat model [16, 17]. The authors have also previously used SHED derived from a patient with Rett syndrome to elucidate the relationship between abnormal DN development and decreased mitochondrial function in vitro [18]. Therefore, SHED are a valuable source of stem cells for DN transplantation and for in vitro disease models.

The aim of this study was to elucidate a relationship between DS and abnormal DN development and function. Here, SHED were prepared from a normal participant and a patient with DS and were then used to examine DS pathology on a cellular level. Our results demonstrate the utility of SHED as a disease model for DS.

## Methods

### Isolation and preparation of SHED

Human exfoliated deciduous teeth were provided by Pediatric Dentistry and Special Need Dentistry at Kyushu University Hospital in Japan. After informed parental consent was obtained, deciduous teeth were collected from a normal participant and a patient with DS at 6 and 14 years of age, respectively. The isolation procedure was completed as previously described [15]. Briefly, the pulp tissue was subjected to an enzymatic dissociation in 3 mg/mL collagenase I (Washington, NJ, USA) and 4 mg/mL dispase II (Wako, Osaka, Japan) for 1 h, and then maintained at 37 °C in a humidified 5% CO_2_ incubator in the Alpha modification of Eagle’s Minimal Essential Medium (α-MEM; Sigma-Aldrich, MO, USA) containing 15% fetal bovine serum (Sigma-Aldrich), 100 μM L-ascorbic acid 2-phosphate (Wako), 2 mM L-glutamine (Life Technologies, NY, USA), 250 μg/mL Fungizone (Life Technologies), 100 U/mL penicillin (Life Technologies), and 100 μg/mL streptomycin (Life Technologies). Cells of not more than 10 passages were used, but Ctrl-SHED and DS-SHED were not always of the same passage.

### Fluorescence in situ hybridization

SHED were treated with 75 mM KCl for 40 min and then fixed with 3:1 ethanol:acetic acid (*v*/v). Fluorescence in situ hybridization (FISH) of chromosome 21 was performed with a chromosome 21 control probe labeling the BAC probe, followed by the standard procedure with green 5-Fluorescein dUTP (CHR21–10-GR; Empire Genomics, NY, USA). Hybridization was performed by denaturing the slides in 70% formamide/2× standard saline citrate, dehydrating the slides with serial ethanol washes, and applying the probe to the slides. Post-hybridization, the slides were washed and stained with 0.1 μg/mL 4′,6-diamidino-2-phenylindole (DAPI; Dojindo, Kumamoto, Japan) to identify nuclei. Fluorescence images were taken with a Zeiss Axio Imager M2 microscope (Zeiss, Oberkochen, Germany) equipped with ApoTome2 (Zeiss).

### Western blotting

Whole-cell lysates were extracted with lysis buffer (62.5 mM Tris-HCl pH 6.8, 2% SDS, 5% β-mercaptoethanol, and 10% glycerol), and the protein concentration was measured using Bradford ULTRA (Novexin, Cambridge, UK). A total of 5 μg of protein was separated by SDS-PAGE and transferred to a polyvinylidene difluoride membrane. After blocking with 5% non-fat milk for 30 min, the membrane was incubated overnight at 4 °C with anti-nestin (1:1000; Millipore, CA, USA) and anti-HSP90 (1:1000; Santa Cruz Biotechnology, CA, USA) antibodies. Membranes were washed and incubated with HRP-conjugated secondary antibody (1:5000; Santa Cruz Biotechnology) for 1 h at room temperature and visualized with ECL prime (GE Healthcare, Buckinghamshire, UK). The chemiluminescent signals were detected and quantified using LAS-1000 pro (Fuji Film, Tokyo, Japan) with Image Gauge software (Fuji Film). HSP90 was used as an internal control. To normalize the nestin expression, the chemiluminescent signal of nestin was divided by the chemiluminescent signal of HSP90.

### DN differentiation

DN differentiation was induced as previously described with minor modifications (brain derived neurotrophic factor [BDNF] was excluded in the second step) [16]. In the first step, 1.5 × 10^5^ SHED were plated onto a 6-well culture plate or glass coverslips coated with 0.01% poly-L-lysine (Sigma-Aldrich), in the same culture medium as described above. They were incubated overnight at 37 °C in the presence of 5% CO_2_, and were then cultured in serum-free Dulbecco’s Modified Eagle’s Medium (DMEM, Sigma-Aldrich) supplemented with 20 ng/mL epidermal growth factor (Sigma-Aldrich), 20 ng/mL basic fibroblast growth factor (Peprotech, NJ, USA), and 1% N2 supplement (Life Technologies) for 2 days at 37 °C, in the presence of 5% CO_2_. In the second step, DMEM was replaced with neurobasal medium (Life Technologies) supplemented with 2% B27 supplement (Life Technologies), 1 mM dibutyryladenosine 3,5-cyclic monophosphate (Sigma-Aldrich), 0.5 mM 3-isobutyl-1-methylxanthine (Sigma-Aldrich), and 200 μM ascorbic acid (Nacalai Tesque, Kyoto, Japan), and cells were incubated for 5 days, at 37 °C, in the presence of 5% CO_2_.

### Immunocytochemistry

**Fig. 1:**
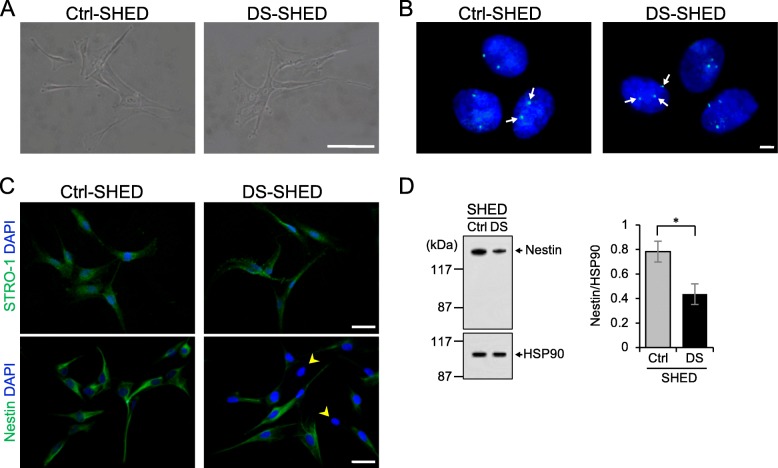
Characterization of SHED isolated from a patient with DS. **a** The morphology of cells in the Ctrl- and DS-SHED was observed using phase-contrast microscopy. Scale bar = 100 μm. **b** Chromosome 21 (white arrows) from the Ctrl- and DS-SHED cells was visualized with FISH. Scale bar = 5 μm. **c** Ctrl- and DS-SHED were stained with anti-STRO-1 (upper panel) and anti-nestin (lower panel) antibodies. The nuclei were counterstained with DAPI. Cells expressing low levels of nestin are indicated with yellow arrows. Scale bar = 50 μm. **d** Nestin expression in Ctrl- and DS-SHED was analyzed using western immunoblotting. The nestin expression was normalized with HSP90. The mean ± SEM from three independent experiments is shown. *P < 0.05

**Fig. 2:**
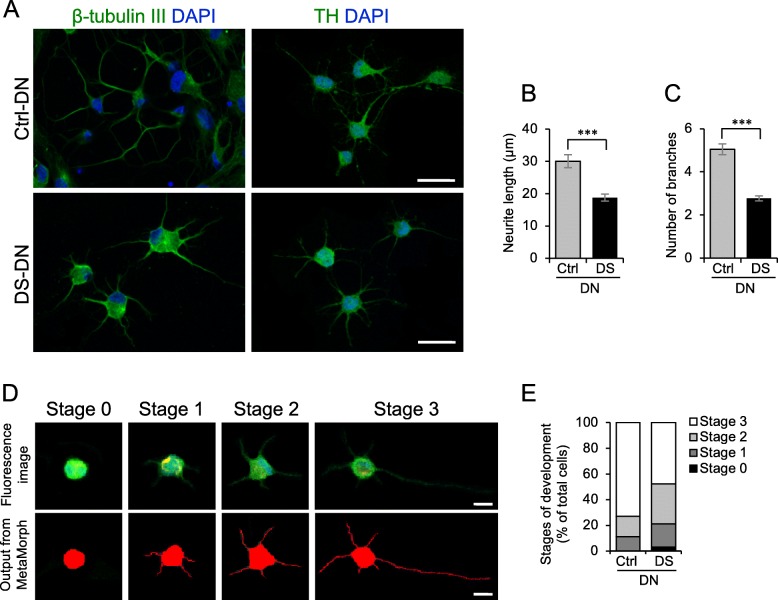
DN differentiation of DS-SHED. **a** Ctrl- and DS-DN cells were observed by immunofluorescence microscopy using anti-β-tubulin III (left panel) and anti-TH (right panel) antibodies. The nuclei were counterstained with DAPI. Scale bar = 50 μm. **b**, **c** Neurite length (**b**) and number of branches (**c**) of Ctrl- and DS-DN cells were measured. The mean ± SEM from 100 cells is shown. ***P < 0.001. **d** DN development was classified into 4 stages. The upper panel shows original TH immunofluorescence images, and the lower panel shows the output from Neurite Outgrowth module of MetaMorph software. **e** A total of 100 differentiated DN were categorized into 4 stages and shown on the graph

**Fig. 3:**
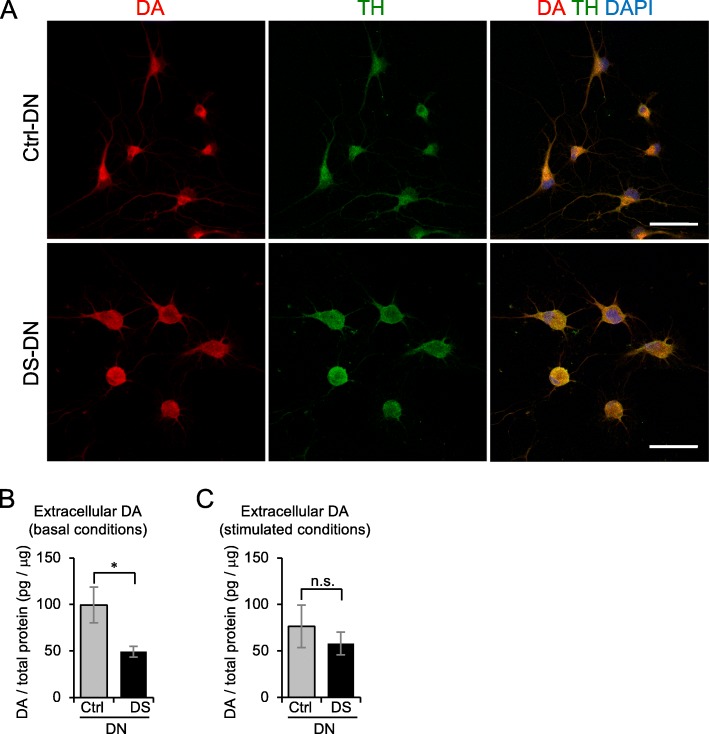
Altered DA secretion in DS-DN. **a** DA expression in Ctrl- and DS-DN cells was observed by immunofluorescence microscopy. Ctrl- and DS-DN cells were stained with anti-DA and anti-TH antibodies, and fluorescence images were captured using the same acquisition settings. Nuclei were counterstained with DAPI; merged images are shown in the right panels. Scale bar = 50 μm. **b**, **c** Extracellular DA amount under basal conditions **b** and glutamate-stimulated conditions **c** were measured by ELISA. The DA amount was normalized with total protein extracted from each cell. Graphs show the mean ± SEM from four experiments. *P < 0.05; n.s., not significant

**Fig. 4:**
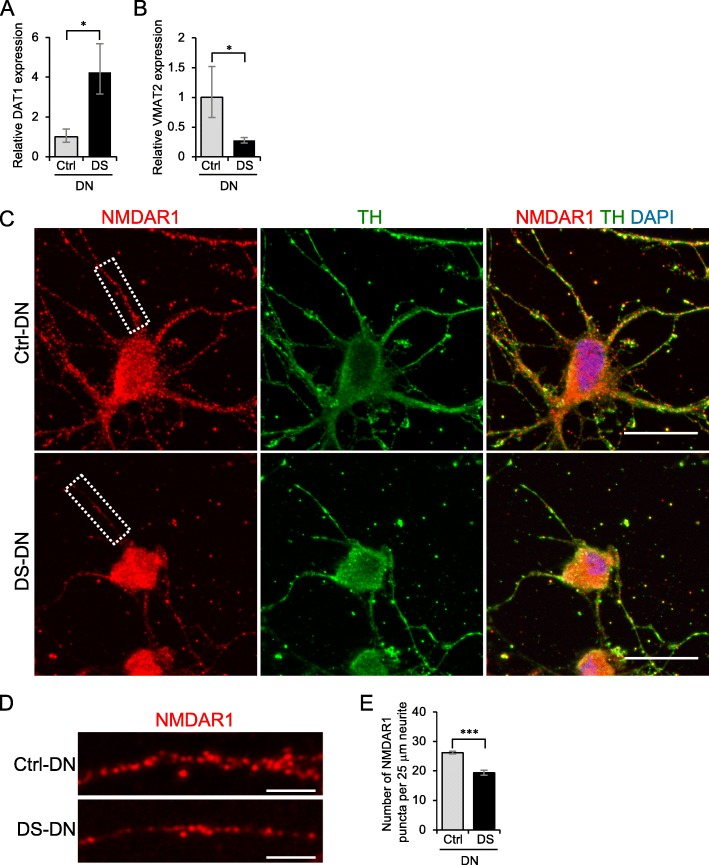
Aberrant expression of molecules involved in DA homeostasis in DS-DN. **a**, **b** The mRNA expression of DAT1 (**a**) and VMAT2 (**b**) was measured by RT-qPCR. The relative expression of each gene was calculated with the 2^-ΔΔCt^ method. Graphs show the mean ± SEM from four experiments. *P < 0.05. **c**-**e** NMDAR1 and TH in Ctrl- and DS-DN cells were observed by immunofluorescence microscopy (**c**). Cells were counterstained with DAPI. Scale bar = 25 μm. Details of the boxed region in (**c**) are shown (**d**). Scale bar = 5 μm. The number of NMDAR1 puncta per 25 μm of neurite was counted (**e**). Graph shows the mean ± SEM of NMDAR1 puncta from 30 neurites. ***P < 0.001

The cells cultured on coverslips were fixed with 4% paraformaldehyde in 0.1 M phosphate buffer (pH 7.4) for 10 min. The cells were permeabilized with 0.1% TritonX-100 for 5 min, then blocked with 2% bovine serum albumin (BSA; Wako) in PBS for 20 min at room temperature. Next, cells were stained with primary antibodies against STRO-1 (1:100; Millipore), nestin (1:250; Millipore), β-tubulin III (1:250; Sigma-Aldrich), tyrosine hydroxylase (TH; 1:100; Millipore), N-methyl-d-aspartate receptor subunit 1 (NMDAR1; 1:100; Millipore), and DA (1:200; Abcam) for 90 min. Following this, cells were incubated with Alexa Fluor secondary antibodies (1:500; Life Technologies) for 1 h at room temperature in the dark. The cells were counterstained with 0.1 μg/mL DAPI (Dojindo) for 5 min, and then mounted with ProLong diamond (Life Technologies). The fluorescence images were taken with Nikon C2 confocal microscope (Nikon, Tokyo, Japan) in Fig. 1c and 3a, with Zeiss LSM700 confocal scanning microscope (Zeiss) in Fig. 2a and d, with Zeiss Axio Imager M2 microscope (Zeiss) equipped with ApoTome2 (Zeiss) in Fig. 4c and d.

### Morphological analysis of DN

For morphological analysis, the TH-immunostained images were analyzed with MetaMorph software (Molecular Devices, CA, USA). A morphological analysis of DN was performed as previously described [19]. Briefly, neurite length and number of branches were measured from 100 TH-positive cells in 20 non-overlapping TH-immunostained images selected randomly from three experiments using the Neurite Outgrowth and Multi-Wavelength Cell Scoring module of MetaMorph software (Molecular Devices). Next, the cells were classified into 4 stages based on neurite length and cell diameter as previously described [19].

### Measurement of NMDAR1 puncta in neurite

To measure the number of NMDAR1 puncta per unit length of neurite, DN were immunostained with anti-NMDAR1 and anti-TH antibodies. Thirty neurites that were in focus and clearly observed were chosen from 10 Ctrl-DN and 12 DS-DN that were positive for both NMDAR1 and TH. These cells were randomly chosen from three experiments. The number of NMDAR1 puncta per 25 μm of neurite was analyzed.

### Extracellular DA measurement

Extracellular DA was measured using a Dopamine Research ELISA kit (BA E-5300, LDN, Nordhorn, Germany) according to the manufacturer’s instructions. SHED were plated at a concentration of 5 × 10^5^ cells per 6-cm dish and differentiated into DN. 500 μl of culture medium was collected to measure extracellular DA. To measure extracellular DA under glutamate stimulated conditions, the cells were treated with 30 μM L-glutamate for 1 min at 37 °C before harvesting the medium. Next, the cell culture medium was centrifuged at 20,400 *g* for 5 min at 4 °C to remove cell debris and immediately stored at − 80 °C until assayed. Subsequently, total protein was extracted from cells using lysis buffer (62.5 mM Tris-HCl pH 6.8 supplemented with 2% SDS, 5% β-mercaptoethanol, and 10% glycerol), and the protein concentration was measured using Bradford ULTRA (Novexin). To normalize the DA amount in each sample, the DA amount was divided by the total protein of that sample.

### RNA extraction and quantitative real-time polymerase chain reaction (RT-qPCR)

Total RNA was extracted from the cells using an RNAeasy Mini Kit (Qiagen, Hilden, Germany). First-strand cDNA was synthesized using a ReverTra Ace qPCR RT Master Mix with gDNA Remover (Toyobo, Osaka, Japan). The sequences of primer sets used in this study were as follows: DAT1: 5’-TGCTGCACAGACACCGTGAG-3′ (forward), 5’-AATGGTCCAGGAGCGTGAAGA-3′ (reverse); VMAT2: 5’-TGAAGAGAGAGGCAACGTCA-3′ (forward), 5’-CGTCTTCCCCACAAACTCAT-3 (reverse); HPRT1: 5’-CCTGGCGTCGTGATTAGTG-3′ (forward), 5’-TCCCATCTCCTTCATCACATC-3′ (reverse). Real-time quantitative PCR was performed using GoTaq qPCR Master Mix (Promega, WI, USA) and analyzed with StepOnePlus Real-Time PCR Systems (Life Technologies). The threshold cycle (Ct) value of HPRT1 was subtracted from the Ct value of the target genes (ΔCt). Statistical analysis was performed using the ΔCt values from four experiments. The relative expressions of the target genes are shown as fold changes determined using the 2^-ΔΔCt^ method.

### Statistical analysis

Values are represented as mean ± standard error of the mean (SEM) from at least three experiments. Two-tailed Student’s *t-*tests were used to compare the Ctrl and DS groups. Differences were considered significant if *p* < 0.05. JMP software (SAS Institute, NC, USA) was used for the statistical analysis.

Methods of figure S1 and S2 are described in Additional file 4.

## Results

### Characteristics of SHED isolated from a patient with DS

We isolated SHED from the deciduous teeth of a child with DS (DS-SHED) and a normal participant (Ctrl-SHED). DS-SHED were spindle-shaped and exhibited a fibroblastic cell morphology that was similar to Ctrl-SHED (Fig. 1a). The presence of 3 copies of chromosome 21 in the nuclei of DS-SHED was verified by FISH (Fig. 1b). Next, analysis of cell proliferation showed similar proliferation of DS-SHED and Ctrl-SHED (Additional file 1: Figure S1). SHED have multi-lineage potential and express mesenchymal and neuronal stem cell markers. To examine the stem cell characteristics of DS-SHED, immunofluorescence staining was performed using antibodies against stem cell markers. Both DS-SHED and Ctrl-SHED expressed STRO-1, a mesenchymal stem cell marker (Fig. 1c, upper panel). In contrast, expression of nestin, a neuronal stem cell marker, was reduced in DS-SHED compared to Ctrl-SHED (Fig. 1c; lower panel; yellow arrows denote cells with weak nestin expression). Western blotting (Fig. 1d) and flow cytometry analysis (Additional file 2: Figure S2) were also used to examine nestin expression and showed reduced nestin expression in DS-SHED compared to Ctrl-SHED.

### Altered differentiation of DS-SHED into DN

We differentiated Ctrl-SHED and DS-SHED into DN, and immunostained these with antibodies to the neuronal marker β-tubulin III and DN marker TH. DN differentiated from DS-SHED (DS-DN) expressed β-tubulin III and TH (Fig. 2a), but neurite length and branching were reduced compared to DN differentiated from Ctrl-SHED (Ctrl-DN). Quantitative analysis also showed that neurite length and branching were reduced in TH-expressing DS-DN compared to Ctrl-DN (Fig. 2b, c). DN development, evaluated in 4 stages according to cell morphology (Fig. 2d; based on a report by Leach et al.) [19], showed that DS-DN development was reduced compared to Ctrl-DN (Fig. 2e).

### Disturbance of DA secretion in DS-DN

A functional analysis of DN was performed by examining DA expression and DA secretion. DA expression was examined by immunostaining cells, which showed that DA expression was present in both Ctrl- and DS-DN (Fig. 3a). Next, DA secretion was examined by measuring extracellular DA and it was observed that extracellular DA of DS-DN was significantly reduced compared with that of Ctrl-DN under basal conditions (*p* = 0.046; Fig. 3b). Although no significant differences between the DS- and Ctrl-DN were observed (*P* = 0.506), extracellular DA of DS-DN was lower than that of Ctrl-DN under glutamate stimulated conditions (Fig. 3c).

### Aberrant expression of molecules involved in DA homeostasis in DS-DN

Possible causes of reduced DA section from DS-DN include the abnormal expression of molecules involved in DA homeostasis, such as dopamine transporter 1 (DAT1) that mediates DA reuptake, vesicular monoamine transporter 2 (VMAT2) that mediates packaging of DA into secretory vesicles, and glutamate receptors. Analysis of DAT1 and VMAT2 mRNA expression showed that DAT1 expression was greater and VMAT2 expression was reduced in DS-DN compared to Ctrl-DN (Fig. 4a, b). Furthermore, expression of NMDAR1, a subunit of glutamate receptor, by immunostaining showed that the number of NMDAR1 puncta per unit length of neurite was reduced in DS-DN compared to Ctrl-DN (Fig. 4c-e).

## Discussion

In this study, various tests on SHED derived from a patient with DS suggest that DN development is reduced and DN function is disturbed in DS. Previous studies have implicated abnormal neuronal cell development in DS based on the examination of induced pluripotent stem cells (iPSCs) and neurospheres derived from the fetal brains of DS patients [20, 21]; however, these studies did not involve the differentiation of stem cells into a specific type of neuronal cell. Thus, the present study focused on DN and is the first to elucidate a relationship between DS and abnormal DN development and function.

Compared to Ctrl-SHED, the expression of the neuronal stem cell marker nestin was reduced in DS-SHED. It has been reported that when iPSCs from a patient with DS are induced to become neural progenitor cells (NPCs), nestin expression, neuronal cell differentiation capacity, neurite length, and synapse formation are reduced compared to controls [20]. In these NPCs, glia markers as well as the differentiation into glia were enhanced over the differentiation into neurons [20]. Although glia were not examined in the present study, because nestin expression was reduced in SHED derived from the patient with DS, it is possible that differentiation into glia was also enhanced, though further investigation is required to confirm this.

A reduction in DA secretion was observed from DS-DN compared to Ctrl-DN. Expression of DAT1, which mediates DA reuptake, was increased and expression of VMAT2, which is involved in packaging DA into secretory vesicles, was decreased in DS-DN compared to Ctrl-DN. An increase in DA reuptake and reduction of DA packaging into secretory vesicles may have led to the observed reduction in extracellular DA of DS-DN. Furthermore, though no significant differences between the DS- and Ctrl-DN were observed, extracellular DA of DS-DN was reduced under glutamate stimulated conditions. The amount of NMDAR1 in neurites was reduced in DS-DN, which could explain the observed reduction in extracellular DA under glutamate stimulated conditions. In addition, DAT1 is important for basal extracellular levels of DA [22] and the dramatic change observed in DAT1 could have caused the cause of the greater difference in DA under basal conditions compared to glutamate stimulated conditions.

DS is caused by an extra copy of chromosome 21 and overexpression of the *DYRK1a* and *DSCR1* genes encoded by chromosome 21 are considered to have particularly important roles in the manifestation of DS symptoms [23, 24]. Overexpression of *DYRK1a* and *DSCR1* in mouse brains is reported to delay differentiation of neuronal precursor cells, causing reduced neuronal development [25]. In our study, neurite length and branching as well as development were reduced in DN derived from DS-SHED. We anticipate that *DYRK1a* and *DSCR1* are involved in this reduced DN development. Further investigation into the expression of these genes and the effects of inhibitors [26, 27] and siRNA in SHED and DN from DS patients are required.

This study used SHED to investigate DS. The stem cell potential of mesenchymal stem cells is reported to change with repeated passages [28]. The present study used SHED of no more than 10 passages. There was no significant difference in cell proliferation and nestin expression between SHED of fewer passages (6 passages) and 10 passages (Additional file 1: Figure S1 and Additional file 2: Figure S2); for this reason, passage-related differences were considered to have a minimal effect on our data. Nevertheless, to increase the utility of the SHED disease model, it will be necessary to determine how many passages of SHED can be used by performing repeated passages and obtaining an accurate understanding of its effect on stem cell marker expression and cell proliferation capacity.

In the present study, we differentiated SHED into DN using a 2-step process based on the method by Fujii et al. [16]; they added brain-derived neurotrophic factor (BDNF) to differentiation media in the second step, but we omitted this addition. Aberrant expression of BDNF is reported in DS patients and mouse models of DS [29–31], suggesting a relationship between neuronal development and BDNF in DS. BDNF is secreted extracellularly and mediates neuronal development and survival. BDNF serves autocrine and paracrine functions [32, 33]. If BDNF exhibits abnormal autocrine function in DS, adding BDNF to the media would conceal DS-SHED pathology. For this reason, BDNF was not added to the media in the present study. It will be necessary to examine BDNF and BDNF receptor expression in future studies to elucidate the involvement of BDNF in DN development of DS.

Fujii et al. reported that *Ngn2* and *Mash1* expression are important as they are activated in the first step of the process that involves differentiation of SHED into early stage DN [16]. In the second step, Fujii et al. speculated that BDNF promotes maturation of early stage DN to DN. When the authors differentiated SHED into DN and performed immunostaining with MAP2 and Tau antibodies, both Ctrl-DN and DS-DN expressed both proteins in whole-cells (Additional file 3: Figure S3). Previous immunostaining studies utilizing mature neuronal cells revealed that the MAP2 antibody stains dendrites, while the Tau antibody stains axons [34, 35]. This suggests that the DN in this study are still developing and have thus not fully matured. This is a limitation of our study. Further studies involving the addition of BDNF are needed to examine synapse formation and other phenomena in mature DN.

## Conclusion

SHED were prepared from a patient with DS and differentiated into DN, revealing abnormal DN development and function. We predict that this DS patient has abnormal DA signaling, but further investigations, such as analyzing cognitive function and DA levels in this DS patient, are necessary. In the future, it will also be necessary to increase the number of patients for analysis to clarify the disturbance in dopaminergic neurodevelopment implicated in the pathophysiology of DS. SHED, which can be prepared noninvasively, offers an effective disease model for this research.

## Additional files

Additional file 1: **Figure S1.** Cell proliferation of SHED in different passage. Ctrl- and DS-SHED were cultured for 24 h and 48 h. The number of cells were counted, and the means ± SEMs from three experiments are shown in the graph. P6; passage 6. P10; passage 10. n.s., not significant. (PDF 10 kb)

Additional file 2: **Figure S2.** Nestin expression in different passages of SHED. Nestin expression in Ctrl- and DS-SHED cells was analyzed with flow cytometry at different passages. P6; passage 6. P10; passage 10. (PDF 186 kb)

Additional file 3: **Figure S3.** Distribution of Tau and MAP2 in DN derived from SHED in this study. Ctrl- and DS-DN were stained with anti-Tau (1:100; Wako) and anti-MAP2 (1:100; Sigma-Aldrich) antibodies. The cells were counterstained with DAPI. The distribution of Tau and MAP2 was observed with Zeiss Axio Imager M2 microscope (Zeiss) equipped with ApoTome2 (Zeiss). Scale bar = 25 μm. (PDF 7737 kb)

Additional file 4: **Supplemental methods.** Methods for Figure S1 and S2. (DOCX 30 kb)

## Abbreviations

DA: Dopamine
DN: dopaminergic neurons
DS: Down Syndrome
NMDAR1: *N*-methyl-D-aspartate receptor subunit NR1
SHED: stem cells from human exfoliated deciduous teeth
TH: tyrosine hydroxylase
