# Supplementary figures and images for: Altered development of dopaminergic neurons differentiated from stem cells from human exfoliated deciduous teeth of a patient with Down syndrome
Source: BMC Neurol. 2018 Aug 31;18:132. doi: 10.1186/s12883-018-1140-2 (PMC6117917; doi:10.1186/s12883-018-1140-2)

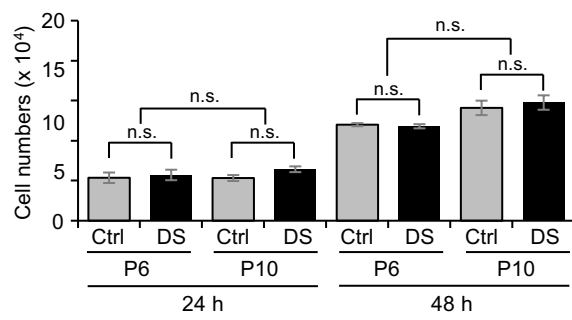

Fig. S1 Pham et al.

Supplement: Supplementary file 1 — Figure S1. Cell proliferation of SHED in different passage. Ctrl- and DS-SHED were cultured for 24 h and 48 h. The number of cells were counted, and the means ± SEMs from three experiments are shown in the graph. P6; passage 6. P10; passage 10. n.s., not significant. (PDF 10 kb) [file 12883_2018_1140_MOESM1_ESM.pdf]

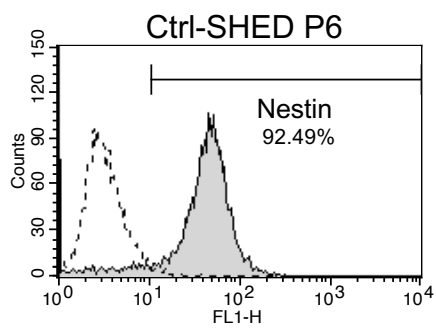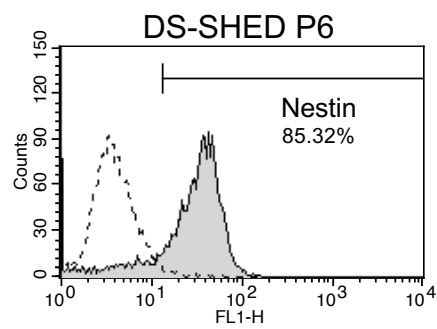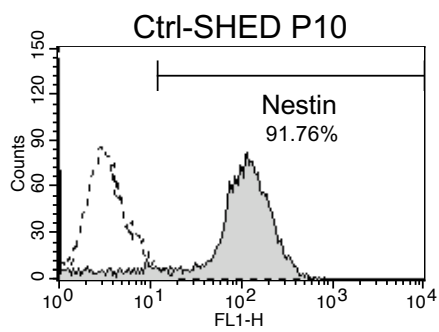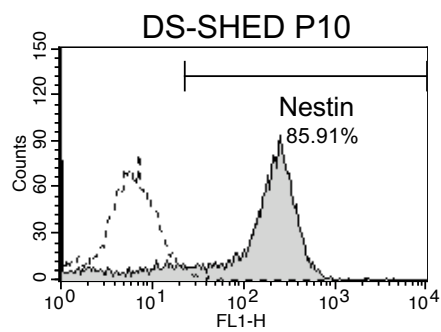

Fig. S2 Pham et al.

Supplement: Supplementary file 2 — Figure S2. Nestin expression in different passages of SHED. Nestin expression in Ctrl- and DS-SHED cells was analyzed with flow cytometry at different passages. P6; passage 6. P10; passage 10. (PDF 186 kb) [file 12883_2018_1140_MOESM2_ESM.pdf]

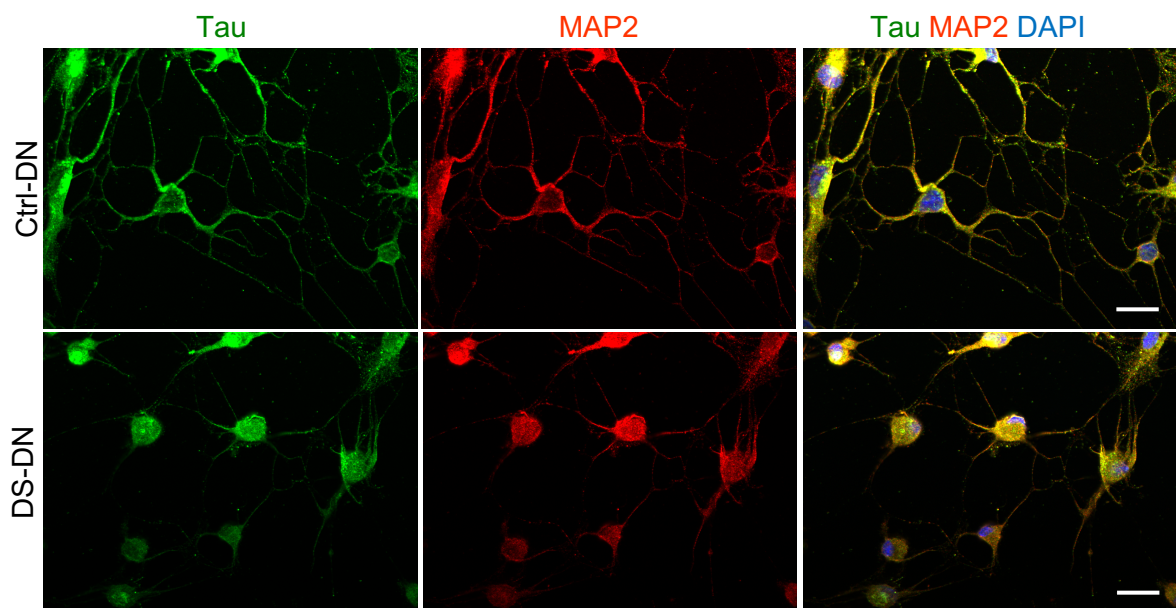

Fig. S3 Pham et al.

Supplement: Supplementary file 3 — Figure S3. Distribution of Tau and MAP2 in DN derived from SHED in this study. Ctrl- and DS-DN were stained with anti-Tau (1:100; Wako) and anti-MAP2 (1:100; Sigma-Aldrich) antibodies. The cells were counterstained with DAPI. The distribution of Tau and MAP2 was observed with Zeiss Axio Imager M2 microscope (Zeiss) equipped with ApoTome2 (Zeiss). Scale bar = 25 μm. (PDF 7737 kb) [file 12883_2018_1140_MOESM3_ESM.pdf]
