# Supplementary material for: Altered development of dopaminergic neurons differentiated from stem cells from human exfoliated deciduous teeth of a patient with Down syndrome
Source: BMC Neurol. 2018 Aug 31;18:132. doi: 10.1186/s12883-018-1140-2 (PMC6117917; doi:10.1186/s12883-018-1140-2)
Supplement: Supplementary file 4 — Supplemental methods. Methods for Figure S1 and S2. (DOCX 30 kb) [file 12883_2018_1140_MOESM4_ESM.docx]

**Supplemental methods**

**Measurement of cell proliferation**

A total of 5 × 10^4^ SHED were seeded onto 6-well culture plates (Corning). After 24 h and 48 h, the cells were collected by trypsinization and stained with 0.4% trypan blue solution (Wako), and subsequently, the cells were counted with a hemocytometer.

**Flowcytometric analysis**

SHED were cultured in a 10-cm culture dish (Corning) at 70% confluency. Cells were treated with Accutase (Nacalai tesque) to detach them from the culture dish. Next, cells were fixed with 2% paraformaldehyde in PBS for 15 min, and permeabilized with 0.2% saponin (Nacalai tesque) for 5 min. Following this, cells were blocked with 1% bovine serum albumin (BSA; Wako) in PBS for 20 min, then cells (1 × 10^6^ per 100 µl) were incubated with anti-nestin (10 µg/mL; Millipore) antibody or mouse IgG_1_ kappa negative control (10 µg/mL; DAKO, Glostrup, Denmark) for 60 min. Subsequently, cells were incubated with Alexa Fluor 488 labeled secondary antibody (1:250; Life Technologies) for 1 h in the dark. Populations of 10,000 cells were analyzed using FACSCalibur (BD Bioscience, CA, USA) and Cell Quest software (BD Bioscience).
